# Glutamate and Synaptic Plasticity Systems and Smoking Behavior: Results from a Genetic Association Study

**DOI:** 10.1371/journal.pone.0038666

**Published:** 2012-06-18

**Authors:** Vanessa Argondizo dos Santos, Jose Miguel Chatkin, Claiton Henrique Dotto Bau, Vanessa Rodrigues Paixão-Côrtes, Ye Sun, Noe Zamel, Katherine Siminovitch

**Affiliations:** 1 School of Medicine, Pontificia Universidade Católica Rio Grande do Sul (PUCRS), Porto Alegre, Brazil; 2 Departament of Genetics, Instituto de Biociências, Universidade Federal do Rio Grande do Sul, Porto Alegre, Brazil; 3 Department of Medicine, University of Toronto, Toronto, Canada; 4 Departments of Immunology and Molecular Genetics, University of Toronto and Samuel Lunenfeld and Toronto General Hospital Research Institutes, Toronto, Ontario, Canada; University of Hong Kong, Hong Kong

## Abstract

Smoking behavior is a multifactorial phenotype with significant heritability. Identifying the specific loci that influence smoking behavior could provide important etiological insights and facilitate the development of treatments to further reduce smoking related mortality. Although several studies pointed to different candidate genes for smoking, there is still a need for replication especially in samples from different countries. In the present study, we investigated whether 21 positive signals for smoking behavior from these studies are replicated in a sample of 531 blood donors from the Brazilian population. The polymorphisms were chosen based on their representativeness of different candidate biologic systems, strength of previous evidence, location and allele frequencies. By genotyping with the Sequenom MassARRAY iPLEX platform and subsequent statistical analysis using Plink software, we show that two of the SNPs studied, in the *SLC1A2* (rs1083658) and *ACTN1* (rs2268983) genes, were associated with smoking behavior in our study population. These genes are involved in crucial aspects of nicotine dependence, glutamate system and synaptic plasticity, and as such, are biologically plausible candidates that merit further molecular analyses so as to clarify their potential role in smoking behavior.

## Introduction

Despite the well documented risks conferred by smoking, cigarette smoking remains highly prevalent in many populations, and continues to be the largest preventable cause of disease and premature death worldwide. It is estimated that there are currently over 1.3 billion smokers globally and that the number will grow to 1.6–1.9 billion by 2025 [1]. Nicotine is the main psychoactive substance in tobacco that underpins smoking addiction, but nicotine dependence (ND) reflects an interplay of neurobiological, environmental and genetic factors [2]. Although the factors influencing smoking behavior are complex, data from large-scale population-based twin and family studies have established significant impact of genetic factors on individual differences in smoking behavior [3]–[5].

Identifying the specific loci that influence smoking behavior could provide important etiological insights and facilitate the development of treatments to further reduce smoking related mortality. Linkage, gene association and genome-wide association studies (GWAS) have identified many chromosomal regions and genes associated with smoking behaviors. Some data have been reproduced in multiple studies, but, in general, results of these analyses have been inconclusive, with few loci replicated in independent studies [6].

Among the genetic studies, candidate and genome-wide association analyses have been the most widely-used strategy to search for genes for nicotine dependence and other smoking-related variables [7]–[11]. One of the strongest genetic associations identified from such studies involves the *CHRNA3-A5-B4* gene cluster at chromosome 15 which encodes NAChR receptors [12]. Another association involves genes related to nicotine metabolism, including the cytochrome *P450 2A6 (CYP2A6)* gene encoding CYP2A6, an enzyme responsible primarily for nicotine metabolism [13]. Associations of smoking behavior with the *TTC12-ANKK1-DRD2* gene cluster [14], [15], which is functionally linked to dopamine in the brain and with genes encoding glutamate receptors (GRIN2B, GRIK2 and GRM8) and transporters (SLC1A2), have also been reported [11], [16]–[18]. This latter association is consistent with increasing evidence for a crucial role of glutamate in the brain reward system and with nicotine roles in enhancing glutamate release and function [19].

These data have, however, been obtained primarily by studies of European and North American populations and thus require replication in other populations before general conclusions can be drawn. We therefore assessed whether 21 of the SNPs identified in prior studies as being associated with smoking behavior are associated with smoking phenotypes also in the Brazilian population. The fact that this sample is composed of Brazilians of European descent permits the inclusion of our data in future meta-analysis.

## Results

Characteristics of the study subject population are presented in Table 1. As shown, the study subjects included 168 smokers and 363 non-smokers. Among the smokers, mean age of smoking onset was 17 years old (±SD 4.0), average number of cigarettes smoked per day was 14 (±SD 11.1), and Fagerstrom Test for Nicotine Dependence (FTDN) levels ranged from medium to severe. Among the non-smokers, there were more male than female subjects and the percentage of subjects completing more than 11 years of schooling was higher than that of the smoking group (39.7% vs. 32.1%, P = 0.002). However, the non-smoking group did not differ from the smoking group in relation to percentage of individuals in stable martial relationships or employment status.

**Table 1:** Demographic characteristics of study subjects.

	Smokers (N = 168)	Non-smokers (N = 363)
Mean (SD)		
Age	37.0 (11.9)	35.0 (11.6)
Cigarettes/day	14.1 (11.1)	-
Age of smoking onset	17.0 (4.0)	-
N (%)		
Male*	86 (51.2)	228 (62.8)
Stable relationship	117 (69.6)	262 (72.2)
Employed	135 (80.4)	317 (87.3)
11+years of schooling**	54 (32.1)	144 (39.7)
FTDN (Moderate/Severe)***	89 (53.6)	-

* P = 0.02.

** P = 0.002.

*** FTND = Fagerstrom Test for Nicotine Dependence: Moderate/High (4–10) vs Low dependence nicotine level scores (0–3).

Results of genetic association data are shown in Table 2, with allele frequencies for cases and controls indicated for all 21 SNPs that passed all quality control criteria, including tests for Hardy–Weinberg equilibrium. These analyses revealed smokers and non-smokers to differ significantly in relation to the respective frequencies of two SNPs, specifically, the rs10836358 SNP at the *SLC1A2* locus (P = 0.047, OR = 1.474) and the rs2268983 SNP at the *ACTN1* locus (P = 0.033, OR = 1.333). With respect to the *SLC1A2* rs10836358 SNP, presence of a T allele was associated with a 1.474 times higher risk of being a smoker, while for the *ACTN1* rs2268983 SNP, the A allele was associated with 1.333 times higher risk for smoking. Since the proteins encoded by these genes have potential influences on nicotine dependence, we also tested for possible associations with FTDN scores, but results were not significant.

**Table 2:** Genetic association data for 21 candidate SNPs for smoking behavior.

Gene	SNP	Chr	Position	Risk allele	F_A	F_U	P	OR
*USH2A*	rs12126638	1	214242319	C	0.222	0.217	0.839	1.034
*GRB14*	rs4423615	2	165146476	G	0.519	0.474	0.182	1.199
*NR3C2*	rs5522	4	149576925	C	0.125	0.111	0.512	1.145
*DRD1*	rs4532	5	174802756	T	0.315	0.343	0.383	1.134
*GRM6*	rs2645339	5	178348669	G	0.494	0.485	0.802	1.034
*GRM8*	rs2237781	7	126462286	A	0.064	0.049	0.319	1.328
*HTR5A*	rs6320	7	154493554	T	0.231	0.281	0.099	1.296
*MSRA*	rs4509385	8	10217606	A	0.491	0.426	0.053	1.299
*CDH23*	rs10999845	10	72910367	A	0.203	0.217	0.602	1.090
*ANKK1*	rs4938015	11	112769854	C	0.345	0.362	0.593	1.078
*BDNF*	rs6265	11	27636492	C	0.196	0.197	0.994	1.001
*LUZP2*	rs10834489	11	24794377	T	0.412	0.391	0.548	1.090
*MICAL2*	rs17477949	11	12175779	T	0.401	0.362	0.236	1.178
SLC1A2	rs10836358	11	35244076	T	0.124	0.173	0.047	1.474
*GRIN2B*	rs7313149	12	13719554	C	0.181	0.158	0.357	1.178
*ACTN1*	rs2268983	14	68478450	A	0.543	0.471	0.033	1.333
*TRIM9*	rs8009082	14	50569094	C	0.198	0.201	0.910	1.019
*CHRNA5*	rs16969968	15	76669980	A	0.347	0.337	0.763	1.044
*CAMKK1*	rs758642	17	3733656	A	0.352	0.347	0.875	1.022
*CABLES1*	rs11082304	18	18974971	T	0.451	0.441	0.753	1.043
*COMT*	rs4680	22	18331271	A	0.447	0.447	0.992	1.001

F_A: risk allele frequency in affected cases.

F_U: risk allele frequency in unaffected controls.

For the remaining 19 SNPs, odds ratio ranged from 1.001 (rs4680 in *COMT*) to 1.328 (rs2237781 in *GRM8*), but no statistically significant associations were detected.

## Discussion

In this study, previously reported associations between smoking behavior and *SLC1A2* and *ACTN1* genomic regions [11], [20], [21] were confirmed in a Brazilian sample. These data provide the first independent confirmation of these associations, suggesting that these two genes may be physiologically and clinically relevant to smoking behavior.

Nicotine is known to produce a variety of rewarding subjective and cognitive effects [22]. However, the neurobiological mechanisms underlying nicotine actions are not well understood, involving not only the direct action of nicotine at acetylcholine receptors, but also other complex neurotransmitter pathways [20], [23]–[26]. Neurochemical studies have demonstrated that nicotine, at concentrations achieved during smoking, can act at presynaptic receptors [27] to enhance the release and function of glutamate. Glutamate is the primary excitatory neurotransmitter in the brain [28]. Alterations in the glutamate uptake system may increase extracellular glutamate levels and corresponding excitotoxicity pathology [29]. Consistently with these findings, alterations in glutamatergic neurotransmission are thought to be involved in several neuropsychiatric disorders, such as schizophrenia, major depressive and bipolar disorders and alcoholism [23], [30].

Association between smoking behavior and the *SLC1A2* genomic region has been previously reported [10], [11]. This is in agreement with a major role for the solute transporter protein family member encoded by *SLC1A2* in clearing glutamate from the extracellular synaptic space in the central nervous system.

Association of the *ACTN1* polymorphism rs2268983 with the number of cigarettes smoked per day has also been previously reported. The *ACTN1* gene belongs to a superfamily that encodes cytoskeletal-related proteins and has been implicated in synaptic plasticity in mammalian brain [31]. Its association with smoking is in keeping with the role of synaptic plasticity in the pathophysiology of addiction [32] and of actin-rich structures, such as dendritic spines in regulation of postsynaptic signaling and synaptic plasticity in the brain [33].

Therefore, the two loci replicated in our association study correspond to highly plausible candidate genes for smoking behavior. The proteins encoded by these genes have potential individual influences on nicotine dependence, and may also have interactive effects with other brain functions. Structural and functional alterations of glutamatergic synapses, for example, may be related to altered synaptic signaling and plasticity, mechanisms that are generally involved in developmental, psychiatric, and neurologic disorders, including nicotine addiction [34].

The present findings, however, have some limitations. The study depends, for example, on the analysis of healthy young Brazilian blood donors, but it is possible that other genes may be more relevant in smokers ascertained from clinical or psychiatric units. A bigger sample size would be desirable. However, the study design and database are suitable for inclusion in future meta-analysis. Also, it is possible that a broader definition of smoking phenotypes might yield different results, since some previous evidences of association were restricted to other phenotypes. It is noteworthy that the fact that positive signals were obtained even with smoking phenotypes that are not exactly the same previously reported might reinforce their position as interesting candidate regions. We could not extend the analysis to other phenotypes for all SNPs considering the lack of statistical power. However, despite these caveats, the partial replication here of other association data and the high biological plausibility of the two candidate genes suggest that both genes should be further evaluated in relation to smoking behavior, with a focus on defining functional variants.

The current findings provide added evidence of the role for genetics in nicotine dependence as is predicted by heritability studies. Our findings also provide incentive for biologic analyses aimed at elucidating the pathways coupling these gene variants to specific smoking behaviors.

## Materials and Methods

### Samples

To carry out a case-control association study, 531 unrelated study subjects were selected from blood donors who attended the São Lucas Hospital of Pontifícia Universidade Catolica do Rio Grande do Sul (PUCRS) in Porto Alegre, Brazil, during the period from March 2009 to August 2010. To be included in this study, subjects had to be Brazilians of European descent between the ages of 18 and 65 years. Former smokers and subjects currently using any smoking cessation medication were excluded from the study. All subjects completed a standardized self-report questionnaire including demographic characteristics and smoking history. Subjects were considered to be smokers if they smoked at least 100 cigarettes over their lifetime and were smoking regularly at the time of the study. Non smokers were defined as individuals who had never smoked or who had smoked less than 100 cigarettes in their lifetime [35].

Level of smoking severity in this sample was assessed using the self-report six-item Fagerström Test for Nicotine Dependence (FTND), a test used widely to provide a quantitative determination of nicotine dependence [36]. Individuals were categorized into 2 levels based on a 0 to 10 scoring scale: low nicotine dependence level (0 to 3) and moderate to severe nicotine dependence level (4 to 10).

The study protocol was approved by the local ethics committee and all subjects gave written informed consent. A summary of demographic characteristics of this sample is provided in Table 1.

### Genotyping

Survey for genes and SNPs was performed in available SNP databases as well as genetics literature. Twenty one SNPs (Table 3) were chosen based on the following criteria: (1) SNPs should be in high priority candidate genes based on the pathophysiology of smoking behaviors and location in replicated chromosomal susceptibility regions by genome scan studies and candidate gene studies; (2) previously reported smoking behavior-associated genomic regions/SNPs; (3) minor allele frequency (MAF) >20% for intronic SNPs and slightly lower (e.g., >10% for coding SNPs in European population; (4) SNPs in the putative promoter, 3'UTR and regulatory domain(s) within the gene(s) as these could potentially impact gene expression and protein stability. Regarding SLC1A2, one of the gene/SNP included, GWAS studies have shown that it is promising candidate gene and has been associated with smoking cessation. However, these original studies reported other SNPs or only mention the genomic region. The specific SNP (rs10836358) was selected based on allele frequency and optimization to the Sequenom MassARRAY iPLEX platform.

**Table 3:** Characteristics of SNPs studied.

Chr	Gene	SNP	Allele	SelectedReference	MainPhenotype
1	USH2A	rs12126638	C/T	^11^	Current smoking
2	GRB14	rs4423615	A/G	^11^	Smoking initiation
4	NR3C2	rs5522	A/G	^7^	Cigarettes/day
5	DRD1	rs4532	C/T	^23^	Cigarettes/day
5	GRM6	rs2645339	C/T	^7^	Cigarettes/day
7	GRM8	rs2237781	A/G	^11^	Smoking initiation
7	HTR5A	rs6320	A/T	^24^	Nicotine dependence
8	MSRA	rs4509385	C/T	^11^	Smoking initiation
10	CDH23	rs10999845	A/G	^11^	Smoking initiation
11	MICAL2	rs17477949	C/T	^11^	Smoking initiation
11	LUZP2	rs10834489	C/T	^11^	Current smoking
11	BDNF	rs6265	A/G	^26^	Current smoking
11	SLC1A2	rs10836358	C/T	^10,11^ *	Current smoking
11	ANKK1	rs4938015	C/T	^13^	Nicotine dependence
12	GRIN2B	rs7313149	C/T	^11^	Smoking initiation
14	TRIM9	rs8009082	A/C	^11^	Smoking initiation
14	ACTN1	rs2268983	C/T	^21^	Cigarettes/day
15	CHRNA5	rs16969968	A/G	^21^	Nicotine dependence
17	CAMKK1	rs758642	C/T	^21^	Cigarettes/day
18	CABLES1	rs11082304	G/T	^21^	Cigarettes/day
22	COMT	rs4680	A/G	^25^	Nicotine dependence

* These studies reported other SNPs or only the genomic region. This specific SNP was selected based on allele frequency and optimization to the Sequenom MassARRAY iPLEX platform.

Genotyping was performed at the Mount Sinai Hospital/University Health Network Gene Profiling Facility at Toronto, Canada. Genomic DNA was extracted from whole blood using the Herrmann and Frischau method [37]. Multiplex SNP assays were designed using SpectroDesigner software, 5–10 ng DNA/subject then amplified by PCR and primer extension products generated using extension primer(s), DNA polymerase, and a deoxynucleotide and di-deoxynucleotide triphosphate cocktail. Amplified DNA was then spotted onto a 384 SpectroChip and analyzed using matrix-assisted laser desorption/ionization time-of-flight (MALDI-TOF) mass spectrometry platform (Sequenom, San Diego, CA).

### Statistical Analysis

To control for genotyping quality, duplicate samples and negative controls were included in the analysis and each SNP had to pass the following criteria: (i) genotype missing rate <10%, (ii) minor allele frequency >1% and (iii) Hardy-Weinberg equilibrium of *P*>10^–4^. Potential confounders (age, gender, marital status, employment status, years of schooling) were included as covariates using a statistical definition (association with both the study factor and outcome for P<0.20) [38]. The results were not significantly influenced by the adjustment. We thus preferred to present unadjusted results for all SNPs in order to make easier the comparability of findings with other studies and inclusion in meta-analyses. Chi-square tests of allele and genotype frequencies in cases and controls and testing of Hardy Weinberg equilibrium for all individual SNPs were performed using Plink (version 1.06) software. Significance level was set at 0.05.

## References

[1] Wipfli H, Samet JM (2009). Global economic and health benefits of tobacco control: part 1.. *Clin Pharmacol Ther* 86, 263–71.

[2] Amos CI, Spitz MR, Cinciripini P (2010). Chipping away at the genetics of smoking behavior.. *Nat Genet* 42, 366–8.

[3] Li MD, Cheng R, Ma JZ, Swan GE (2003). A metaanalysis of estimated and environmental effects on smoking behavior in male and female adult twins.. *Addiction* 98, 23–31.

[4] Vink JM, Beem AL, Posthuma D, Neale MC, Willemsen G (2004). Linkage analysis of smoking initiation and quantity in Dutch sibling pairs.. *Pharmacogenomics J* 4, 274–82.

[5] Vink JM, Willemsen G, Boomsma DI (2005). Heritability of smoking initiation and nicotine dependence.. *Behav Genet* 35, 397–406.

[6] Li MD (2008). Identifying susceptibility loci for nicotine dependence: 2008 update based on recent genome-wide linkage analyses.. *Hum Genet* 123, 119–31.

[7] Berrettini W, Yuan X, Tozzi F, Song K, Francks C (2008). Alpha-5/alpha-3 nicotinic receptor subunit alleles increase risk for heavy smoking.. *Mol Psychiatry* 13, 368–73.

[8] Bierut LJ, Madden PA, Breslau N, Johnson EO, Hatsukami D (2007). Novel genes identified in a high-density genome wide association study for nicotine dependence.. *Hum Mol Genet* 16, 24–35.

[9] Liu QR, Drgon T, Walther D, Johnson C, Poleskaya O, Hess J, Uhl GR (2005). Pooled association genome scanning: validation and use to identify addiction vulnerability loci in two samples.. *Proc Natl Acad Sci U S A* 102, 11864–9.

[10] Uhl GR, Liu QR, Johnson C, Walther D, Rose JE (2008). Molecular genetics of successful smoking cessation: convergent genome-wide association study results.. *Arch Gen Psychiatry* 65, 683–93.

[11] Vink JM, Smit AB, Geus ECJ, Sullivan P, Willemsen G (2009). Genome-wide association study of smoking initiation and current smoking.. *Am J Med Genet* 84, 367–79.

[12] Liu JZ, Tozzi F, Waterworth DM, Pillai SG, Muglia P (2010). Meta-analysis and imputation refines the association of 15 q25 with smoking quantity (Ox-GSK consortium).. *Nat Genet* 42, 436–40.

[13] Thorgeirsson TE, Guddjartsson DF, Surakka I, Vink JM, Amin N (2010). Sequence variants at CHRNB3-CHRNA6 and CYP2A6 affect smoking behavior(ENGAGE consortium).. *Nat Genet* 42, 448–53.

[14] Ducci F, Kaakinen M, Pouta A, Hartikainen AL, Veijola J (2011). TTC12-ANKK1-DRD2 and CHRNA5-CHRNA3-CHRNB4 influence different pathways leading to smoking behavior from adolescence to mid-adulthood.. *Biol Psychiatry* 69, 650–60.

[15] Gelernter J, Yu Y, Weiss R, Brady K, Panhuysen C (2006). Haplotype spanning TTC12 and ANKK1, flanked by the DRD2 and NCAM1 loci, is strongly associated to nicotine dependence in two distinct American populations.. *Hum Mol Genet* 15, 3498–507.

[16] Flatscher-Bader T, Zuvela N, Landis N, Wilce PA (2008). Smoking and alcoholism target genes associated with plasticity and glutamate transmission in the human ventral tegmental area.. *Hum Mol Genet* 17, 38–51.

[17] Jackson A, Nesic J, Groombridge C, Clowry O, Rusted J (2009). Differential involvement of glutamatergic mechanisms in the cognitive and subjective effects of smoking.. *Neuropsychopharmacology* 34, 257–65.

[18] Liechti ME, Markou A (2008). Role of the glutamatergic system in nicotine dependence : implications for the discovery and development of new pharmacological smoking cessation therapies.. *CNS Drugs* 22, 705–24.

[19] Nesic J, Duka T, Rusted JM, Jackson A (2011). A role for glutamate in subjective response to smoking and its action on inhibitory control.. *Psychopharmacology* 216, 29–42.

[20] Watkins SS, Koob GF, Markou A (2000). Neural mechanisms underlying nicotine addiction: acute positive reinforcement and withdrawal.. *Nicotine Tob Res* 2, 19–37.

[21] Caporaso N, Gu F, Chatterjee N, Sheng-Chih J, Yu K (2009). Genome-wide and candidate gene association study of cigarette smoking behaviors.. *PLoS One* 4, e4653.

[22] Levin ED, McClernon FJ, Rezvani AH (2006). Nicotinic effects on cognitive function: behavioral characterization, pharmacological specification, and anatomic localization.. *Psychopharmacology* 184, 523–39.

[23] Novak G, LeBlanc M, Zai C, Shaikh S, Renou J (2010). Association of polymorphisms in the BDNF, DRD1 and DRD3 genes with tobacco smoking in schizophrenia.. *Ann Hum Genet* 74, 291–8.

[24] Saccone SF, Hinrichs AL, Saccone NL, Chase GA, Konvicka K (2007). Cholinergic nicotinic receptor genes implicated in a nicotine dependence association study targeting 348 candidate genes with 3713 SNPs.. *Hum Mol Genet* 16, 36–49.

[25] Beuten J, Payne TJ, Ma JZ, Li MD (2006). Significant association of catechol-O-methyltransferase (COMT) haplotypes with nicotine dependence in male and female smokers of two ethnic populations.. *Neuropsychopharmacology* 31, 675–84.

[26] Lang UE, Sander T, Lohoff FW, Hellweg R, Bajbouj M (2007). Association of the met66 allele of brain-derived neurotrophic factor (BDNF) with smoking.. *Psychopharmacology* 190, 433–9.

[27] McGehee DS, Heath MJ, Gelber S, Devay P, Role LW (1995). Nicotine enhancement of fast excitatory synaptic transmission in CNS by presynaptic receptors.. *Science* 269, 1692–6.

[28] Monaghan DT, Bridges RJ, Cotman CW (1989). The excitatory amino acid receptors: their classes, pharmacology, and distinct properties in the function of the central nervous system.. *Ann Rev Pharmacol Toxicol* 29, 365–402.

[29] Takahashi M, Billups B, Rossi D, Sarantis M, Hamann M (1997). The role of glutamate transporters in glutamate homeostasis in the brain.. *J Exp Biol* 200, 401–9.

[30] Vengeliene V, Bilbao A, Molander A, Spanagel R (2008). Neuropharmacology of alcohol addiction.. *Br J Pharmacol* 154, 299–315.

[31] Walikonis RS, Oguni A, Khorosheva EM, Jeng CJ, Asuncion FJ (2001). Densin-180 forms a ternary complex with the (alpha)-subunit of Ca2+/calmodulin-dependent protein kinase II and (alpha)-actinin.. *J Neurosci* 21, 423–33.

[32] Robinson TE, Kolb B (2004). Structural plasticity associated with exposure to drugs of abuse.. *Neuropharmacology* 47, 33–46.

[33] Harris KM (1999). Structure, development, and plasticity of dendritic spines.. *Curr Opin Neurobiol* 9, 343–8.

[34] van Spronsen M, Hoogenraad CC (2010). Synapse pathology in psychiatric and neurologic disease.. *Curr Neurol Neurosci Rep* 10, 207–14.

[35] C (1994). DC - Centers for Disease Control and Prevention. Cigarette Smoking Among Adults – United States, 1992, and Changes in the Definition of Current Cigarette Smoking.. *MMWR* 43, 342–6.

[36] Heatherton TF, Kozlowski LT, Frecker RC, Fagerström KO (1991). The Fagerström Test for Nicotine Dependence: a revision of the Fagerström Tolerance Questionnaire.. *Brit J Addict* 86, 1119–27.

[37] Herrmann BG, Frischauf AM (1987). Isolation of genomic DNA.. *Methods Enzymol* 152, 180–3.

[38] Maldonado G, Greenland S (1993). Simulation study of confounder selection strategies.. *Am J Epidemiol* 138, 923–36.

